# Weak convergence to isotropic complex $S\alpha S$ random measure

**DOI:** 10.1186/s13660-017-1505-x

**Published:** 2017-09-21

**Authors:** Jun Wang, Yunmeng Li, Liheng Sang

**Affiliations:** 1 School of Mathematics and Finance, Chuzhou University, Chuzhou, 239000 People’s Republic of China; 2 Department of Mathematics, Anhui Normal University, Wuhu, 241000 People’s Republic of China

**Keywords:** weak convergence, isotropic, complex $S\alpha S$ random measure, Poisson processes

## Abstract

In this paper, we prove that an isotropic complex symmetric *α*-stable random measure ($0<\alpha<2$) can be approximated by a complex process constructed by integrals based on the Poisson process with random intensity.

## Introduction

Let $\alpha\in(0,2)$, $X_{1}$ and $X_{2}$ be real random variables defined on the same probability space. A complex random variable $X=X_{1}+i X_{2}$ is called symmetric *α*-stable (in short $S\alpha S$) if the random vector $(X_{1},X_{2})$ is $S\alpha S$ in $\mathbb{R}^{2}$, where *i* is an imaginary unit. Furthermore, a complex $S\alpha S$ random variable $X=X_{1}+i X_{2}$ is isotropic if and only if there exist two independent and identically distributed (in short for i.i.d.) zero mean normal random variables $G_{1}$, $G_{2}$ and an $\alpha/2$-stable random variable *A*, independent of $(G_{1}, G_{2})$, totally skewed to the right, such that $(X_{1}, X_{2})$ is sub-Gaussian with underlying vector $(G_{1}, G_{2})$, that is to say,

$$(X_{1}, X_{2})\stackrel{d}{=}(\sqrt{A}G_{1}, \sqrt{A}G_{2}), $$

where $\stackrel{d}{=}$ denotes the finite-dimensional distribution equality. Let *G* be a complex Gaussian random variable satisfying $G=G_{1}+iG_{2}$, where $G_{1}$, $G_{2}$ are i.i.d. zero mean normal random variables. Thus, every complex isotropic $S\alpha S$ random variable *X* with $\alpha<2$ can be denoted by $X=\sqrt{A}G$.

Much of present works mainly focus on real-valued random variables; however, in Samorodnitsky and Taqqu [1], Cohen [2, 3], Benassi et al. [4], they encounter an important class of real-valued stable processes, which are defined in terms of integral with respect to complex $S\alpha S$ random measure. These show that complex random measure is very important in theory. Next, we introduce a series of processes approximating to different random measures based on the primary works of Stroock [5]. Finally, we construct a process to approximate to complex-valued random measure.

Let $\{N(t), t\in[0,\infty)\}$ be a standard Poisson process. For all $n\geq1$, define a process $U_{n}=\{U_{n}(t), t\in[0,\infty)\}$ by

$$U_{n}(t)=\sqrt{n} \int_{0}^{t}(-1)^{N(ns)}\,ds, \quad t\geq0. $$

Stroock [5] proved that when $n\rightarrow\infty$, the law of $U_{n}$ converges weakly in the Banach space $\mathcal{C}([0,T])$ of continuous functions on $[0,T]$, to a Wiener measure. Bardina and Jolis [6] proved that

$$U_{n}(s,t)= {n} \int_{0}^{t} \int_{0}^{s}\sqrt{xy}(-1)^{N(\sqrt{n}x,\sqrt{n}y)}\,dx\,dy $$

converges in law in the space of continuous functions on $\mathcal{C} ([0,1]^{2})$, the space of continuous functions on $[0,1]\times[0,1]$, as $n\rightarrow\infty$, to the ordinary Brownian sheet, where $\{N(x,y),(x,y)\in \mathbb{R}_{+}^{2}\}$ is a standard Poisson process in the plane. Let $\{N_{\alpha}(t), t\in[0,\infty)\}$ be a Poisson process with random intensity. Under this condition, when $n\rightarrow\infty$, Dai and Li [7] proved that the process $X_{n}=\{X_{n}(t)\}$ defined by

$$X_{n}(t)=\sqrt{n} \int_{0}^{t}(-1)^{N_{\alpha}(ns)}\,ds, \quad0\leq t \leq1, $$

converges weakly in $\mathcal{C}([0,1])$ to a sub-Gaussian process $X_{\alpha}=\{\sqrt{A_{1}}W(t), t\in[0,1]\}$, where $\{W(t), t\in[0,1]\}$ is a standard Brownian motion and $A_{1}$ is a random variable with the same law as *A*. They also proved under certain conditions that

$$X_{n}(s,t)= {n} \int_{0}^{t} \int_{0}^{s}\sqrt{xy}(-1)^{N_{\alpha}(\sqrt{n}x,\sqrt {n}y)}\,dx\,dy, \quad0 \leq t\leq1, $$

converges weakly in $\mathcal{C}([0,1]^{2})$ to a two-parameter standard sub-Gaussian process, where $\{N_{\alpha}(x, y); x,y \in[0,\infty)\}$ is a two-parameter Poisson process with random intensity.

On the other hand, on approximation to complex Brownian motion, Bardina [8] considered the process $U_{n}^{\theta}=\{U_{n}^{\theta}(t), t\in[0,\infty)\}$ defined by

$$U_{n}^{\theta}(t)=\sqrt{n} \int_{0}^{2t}e^{i\theta{N(ns)}}\,ds, \quad t\geq0, $$

where *i* is an imaginary unit. Bardina proved that if $\theta\in(0,\pi)\cup(\pi,2\pi)$, when $n \rightarrow\infty$, $P_{n}^{\theta}$ the image law of $U_{n}^{\theta}$ in the Banach space $\mathcal{C}([0,T],\mathbb{C})$ converges weakly to the law of a complex Brownian motion in $\mathcal{C}([0,T],\mathbb{C})$. When $\theta=\pi$, $P_{n}^{\theta}$, converges weakly to the law of $\sqrt{2}W(t)$, where $\{W(t), t\in [0,T]\} $ is a standard Brownian motion.

Inspired by the above works, in this paper, we will prove similar results about an isotropic complex $S\alpha S$ random variable. Define

1 $$ X_{n}^{\theta}(t)=\sqrt{n} \int_{0}^{2t}e^{i\theta N_{\alpha}(nr)}\,dr, \quad 0\leq t \leq1 , $$

where $\{N_{\alpha}(t), t\in[0,\infty)\}$ is a Poisson process with random intensity $\frac{1}{A}$. In the trivial case, when $\theta=0$, the process $X_{n}^{\theta}(t)$ is deterministic, and when *n* tends to infinity, $X_{n}^{\theta}(t)$ goes to infinity. When $\theta=\pi$, the process $X_{n}^{\theta}(t)$ is real and (1) becomes

2 $$ X_{n}^{\theta}(t)=\sqrt{n} \int_{0}^{2t}(-1)^{N_{\alpha}(nr)}\,dr, \quad0\leq t \leq1 , $$

this case was studied by Dai and Li [7].

We will prove under certain conditions, when $\theta\in(0,\pi)\cup(\pi,2\pi)$, that the law of $X_{n}^{\theta}$ converges weakly in $\mathcal{C}([0,1],\mathbb{ C})$ to the law of isotropic complex $S\alpha S$ random measure.

The rest of the paper is organized as follows. First we give preliminaries and the main result, then we present some lemmas, including proving the tightness and identification of the limit law, to prove the main result.

## Preliminaries and the main result

Now we give some preliminary definitions and the main result.

### Definition 2.1

[7]

Let $(\Omega_{1}, \mathcal{F}_{1}, P_{1})$ be a probability space. Suppose that *A* is a nonnegative random variable on the probability space $(\Omega_{2}, \mathcal{F}_{2}, P_{2})$. Let $(\Omega, \mathcal{F}, P)=(\Omega_{1}\times\Omega_{2}, \mathcal {F}_{1}\times\mathcal{F}_{2}, P_{1}\times P_{2})$ be the underlying probability space of this paper. Let $N=\{N(t),t\geq0\}$ be a counting process on $(\Omega, \mathcal{F}, P)$ satisfying the following assumptions:

- When $A>0$ is given, *N* is a Poisson process on $(\Omega_{1}, \mathcal{F}_{1}, P_{1})$ with intensity $\frac{1}{A}$;
- When $A=0$, $N=0$ a.s.

Then we call *N* as the Poisson process with random intensity $\frac{1}{A}$.

Throughout this paper, we define the Poisson process $\{N_{\alpha}(t), t\geq0\}$ with random intensity $\frac{1}{A}$, we assume that *A* is a strictly $\frac{\alpha }{2}$-stable random variable with respect to $(\Omega_{2}, \mathcal{F}_{2}, P_{2})$, totally skewed to the right, with Laplace transform given by

$$ E\exp\{-\lambda A\}= \exp \bigl\{ -\lambda^{\frac{\alpha}{2}} \bigr\} . $$

Considering the sequences $X_{n}^{\theta}$ defined by (1), we have the following theorem.

### Theorem 2.1

*Let the process* $\{X_{n}^{\theta}(t), 0\leq t \leq1\}$, *considering* $P_{n}^{\theta}$ *the image law of* $X_{n}^{\theta}$, *in the Banach space* $\mathcal{C}([0,1], \mathbb{C})$. *Then*, *if* $\theta\in(0,\pi)\cup(\pi,2\pi)$, *when* *n* *tends to infinity*, $P_{n}^{\theta}$ *converges weakly to the law in* $\mathcal{C}([0,1],\mathbb{C})$ *of isotropic complex* $S\alpha S$ *random measure* $\{X(t)=\sqrt{A_{1}}G(t)\}$, $0\leq t\leq1$, *where* $\{G(t)\}$ *is complex Gaussian measure*, $A_{1}$ *is a random variable with the same law as* *A*.

The main idea of the proof of the theorem is that, when $N_{\alpha}=\{N_{\alpha}(t), t\in[0,\infty)\}$ is a Poisson process with random intensity *A*, the process $X_{n}^{\theta}/\sqrt{A}$ converges weakly to complex Brownian motion independent of *A*. Then, according to the idea of [8], we need to check that if $\theta\in(0,\pi)\cup(\pi,2\pi)$, the family $P_{n}^{\theta}$ is tight and the law of all possible weak limits of $P_{n}^{\theta}$ is the law of a complex Brownian motion.

We split the proof of Theorem 2.1 into two parts. We first prove the tightness of the process $X_{n}^{\theta}$ and then identify the limit law of the process $X_{n}^{\theta}$.

In this paper, *K* denotes a positive constant independent of *n*, it may change value from one expression to another.

### Proof of tightness

We give an auxiliary process $Y_{n}^{\theta}=\{Y_{n}^{\theta}(t),t\in[0,T]\}$, $T>0$, denoted by

$$ Y_{n}^{\theta}(t)=\mathbf{1}_{\{A>0\}}\sqrt{ \frac{n}{A}} \int _{0}^{2t}e^{i\theta N_{\alpha}(nr)}\,dr. $$

For any $n\geq1$, we have

3 $$ \mathbb{P} \bigl(X_{n}^{\theta}(t)= \sqrt{A}Y_{n}^{\theta}(t), t\in[0,1] \bigr)=1. $$

Since $\mathbb{P}(A=0)=0$.

Denote

$$ I_{1,n}^{\theta}(t)= \biggl[\mathbf{1}_{\{A>0\}}\sqrt{ \frac{n}{A}} \biggl[ \int_{0}^{2t}\cos \bigl(\theta N_{\alpha}(nr) \bigr)\,dr \biggr] \biggr]=\operatorname {Re} \bigl[Y_{n}^{\theta}(t) \bigr], $$

and

$$ I_{2,n}^{\theta}(t)= \biggl[\mathbf{1}_{\{A>0\}}\sqrt{ \frac{n}{A}} \biggl[ \int_{0}^{2t}\sin \bigl(\theta N_{\alpha}(nr) \bigr)\,dr \biggr] \biggr]=\operatorname {Im} \bigl[Y_{n}^{\theta}(t) \bigr]. $$

### Lemma 2.1

*There exists a constant* *K* *such that*, *when* $\theta\in(0,\pi)\cup(\pi ,2\pi)$, *for any* $s,t \in[0,1]$ *and* $n>0$,

$$ \mathbb{E} \bigl[I_{1,n}^{\theta}(t)-I_{1,n}^{\theta}(s) \bigr]^{4}+\mathbb {E} \bigl[I_{2,n}^{\theta}(t)-I_{2,n}^{\theta}(s) \bigr]^{4}\leq K(t-s)^{2}. $$

### Proof

Without loss of generality, we assume $s< t$. Then

$$\begin{aligned} & \mathbb{E} \bigl[I_{1,n}^{\theta}(t)-I_{1,n}^{\theta}(s) \bigr]^{4}+\mathbb {E} \bigl[I_{2,n}^{\theta}(t)-I_{2,n}^{\theta}(s) \bigr]^{4} \\ &\quad =\mathbb{E} \Biggl[\mathbf{1}_{\{A>0\}}\frac{n^{2}}{A^{2}} \int _{[2s,2t]^{4}}\bigotimes_{j=1}^{4} \cos \bigl(\theta N_{\alpha}(nr_{j}) \bigr)\,dr_{1} \,dr_{2}\,dr_{3}\,dr_{4} \\ &\qquad{}+\mathbf{1}_{\{A>0\}}\frac{n^{2}}{A^{2}} \int _{[2s,2t]^{4}}\bigotimes_{j=1}^{4} \sin \bigl(\theta N_{\alpha}(nr_{j}) \bigr)\,dr_{1} \,dr_{2}\,dr_{3}\,dr_{4} \Biggr] \\ &\quad =:B_{1}+B_{2}, \end{aligned}$$

where

$$ \begin{gathered} B_{1}=\frac{1}{2}\mathbb{E} \Biggl[ \mathbf{1}_{\{A>0\}}\frac{n^{2}}{A^{2}} \int _{[2s,2t]^{4}}\bigotimes_{j=1}^{2} \cos \bigl(\theta \bigl(N_{\alpha}(nr_{2j})-N_{\alpha}(nr_{2j-1}) \bigr) \bigr)\bigotimes_{l=1}^{4} dr_{l} \Biggr], \\ B_{2}=\frac{1}{2}\mathbb{E} \Biggl[\mathbf{1}_{\{A>0\}} \frac{n^{2}}{A^{2}} \int _{[2s,2t]^{4}}\bigotimes_{j=1}^{2} \cos \bigl(\theta \bigl(N_{\alpha}(nr_{2j})+N_{\alpha}(nr_{2j-1}) \bigr) \bigr)\bigotimes_{l=1}^{4} dr_{l} \Biggr]. \end{gathered} $$

Using the independence increments of the Poisson process, we obtain

$$\begin{aligned} B_{1} &= \frac{1}{2}\mathbb{E} \Biggl[\mathbf{1}_{\{A>0\}} \frac{n^{2}}{A^{2}} \int _{[2s,2t]^{4}}\bigotimes_{j=1}^{2} \mathbb{E} \bigl[ \cos \bigl(\theta \bigl(N_{\alpha}(nr_{2j})-N_{\alpha}(nr_{2j-1}) \bigr) \bigr)\vert A \bigr]\bigotimes_{l=1}^{4} \,dr_{l} \Biggr] \\ & \leq12\mathbb{E} \biggl[\mathbf{1}_{\{A>0\}}\frac{n^{2}}{A^{2}} \int _{2s}^{2t} \int_{2s}^{r_{2}}\mathbb{E} \bigl[\cos \bigl(\theta \bigl(N_{\alpha}(nr_{2})-N_{\alpha}(nr_{1}) \bigr) \bigr)\vert A \bigr]\,dr_{1}\,dr_{2} \\ &\quad{} \times \int_{2s}^{2t} \int_{2s}^{r_{4}}\mathbb{E} \bigl[\cos \bigl(\theta \bigl(N_{\alpha}(nr_{4})-N_{\alpha}(nr_{3}) \bigr) \bigr)\vert A \bigr]\,dr_{3}\,dr_{4} \biggr] \\ & \leq12\mathbb{E} \biggl[\mathbf{1}_{\{A>0\}}\frac{n^{2}}{A^{2}} \int _{2s}^{2t} \int_{2s}^{r_{2}} \bigl\Vert \mathbb{E} \bigl[\cos \bigl(\theta \bigl(N_{\alpha}(nr_{2})-N_{\alpha}(nr_{1}) \bigr) \bigr)\vert A \bigr] \bigr\Vert \,dr_{1}\,dr_{2} \\ &\quad{} \times \int_{2s}^{2t} \int_{2s}^{r_{4}} \bigl\Vert \mathbb {E} \bigl[\cos \bigl(\theta \bigl(N_{\alpha}(nr_{4})-N_{\alpha}(nr_{3}) \bigr) \bigr)\vert A \bigr] \bigr\Vert \,dr_{3}\,dr_{4} \biggr], \end{aligned}$$

where $\Vert \cdot \Vert $ denotes the modulus of the complex number. It is easy to obtain

$$ \bigl\Vert \mathbb{E} \bigl[\cos \bigl(\theta \bigl(N_{\alpha}(nr_{2})-N_{\alpha}(nr_{1}) \bigr) \bigr)\vert A \bigr] \bigr\Vert \leq e^{-\frac{n}{A}(r_{2}-r_{1})(1-\cos\theta)} $$

and

$$ \bigl\Vert \mathbb{E} \bigl[\cos \bigl(\theta \bigl(N_{\alpha}(nr_{4})-N_{\alpha}(nr_{3}) \bigr) \bigr)\vert A \bigr] \bigr\Vert \leq e^{-\frac{n}{A}(r_{4}-r_{3})(1-\cos\theta)}. $$

Then

$$\begin{aligned} B_{1} &\leq12\mathbb{E} \Biggl[\mathbf{1}_{\{A>0\}} \frac{n^{2}}{A^{2}} \int _{2s}^{2t} \int_{2s}^{r_{2}} \int_{2s}^{2t} \int_{2s}^{r_{4}} e^{-\frac{n}{A}(r_{2}-r_{1})(1-\cos\theta)} e^{-\frac{n}{A}(r_{4}-r_{3})(1-\cos\theta)}\bigotimes_{l}^{4} dr_{l} \Biggr] \\ &\leq12\mathbb{E} \biggl[\mathbf{1}_{\{A>0\}}\frac{n^{2}}{A^{2}} \frac {4A^{2}(t-s)^{2}}{(1-\cos\theta)^{2}n^{2}} \biggr] \\ &=\frac{48}{(1-\cos\theta)^{2}}(t-s)^{2}. \end{aligned}$$

Next we calculate $B_{2}$. Considering the fact that

$$\begin{aligned} & \bigl[\cos(x_{4}+x_{3})\cos(x_{2}+x_{1}) \bigr] \\ &\quad = \bigl[\cos \bigl((x_{4}-x_{3})+(x_{2}-x_{1})+(2x_{3}+x_{1}-x_{2}) \bigr)\cos(x_{2}+x_{1}) \bigr] \\ &\quad = \bigl[\cos \bigl((x_{4}-x_{3})+(x_{2}-x_{1}) \bigr) \bigr] \bigl[\cos(2x_{3}+x_{1}-x_{2}) \cos(x_{2}+x_{1}) \bigr] \\ &\qquad{}- \bigl[\sin \bigl((x_{4}-x_{3})+(x_{2}-x_{1}) \bigr) \bigr] \bigl[\sin(2x_{3}+x_{1}-x_{2}) \cos(x_{2}+x_{1}) \bigr] \\ &\quad \leq \bigl\vert \bigl[\cos \bigl((x_{4}-x_{3})+(x_{2}-x_{1}) \bigr) \bigr] \bigr\vert + \bigl\vert \bigl[\sin \bigl((x_{4}-x_{3})+(x_{2}-x_{1}) \bigr) \bigr] \bigr\vert \\ &\quad \leq \bigl[ \bigl\vert \bigl[\cos(x_{4}-x_{3}) \bigr] \bigr\vert + \bigl\vert \bigl[\sin (x_{4}-x_{3}) \bigr] \bigr\vert \bigr] \times \bigl[ \bigl\vert \bigl[\cos(x_{2}-x_{1}) \bigr] \bigr\vert + \bigl\vert \bigl[\sin(x_{2}-x_{1}) \bigr] \bigr\vert \bigr]. \end{aligned}$$

By the independence increments of the Poisson process, we have

$$\begin{aligned} B_{2}&=\frac{1}{2}\mathbb{E} \Biggl[\mathbf{1}_{\{A>0\}} \frac{n^{2}}{A^{2}} \int _{[2s,2t]^{4}}\bigotimes_{j=1}^{2} \cos \bigl(\theta \bigl(N_{\alpha}(nr_{2j})+N_{\alpha}(nr_{2j-1}) \bigr) \bigr)\bigotimes_{l=1}^{4} dr_{l} \Biggr] \\ &\leq\frac{1}{2}\mathbb{E} \Biggl[\mathbf{1}_{\{A>0\}} \frac{n^{2}}{A^{2}} \int_{[2s,2t]^{4}} \bigl[ \bigl\vert \mathbb{E} \bigl[\cos \bigl( \theta \bigl(N_{\alpha}(nr_{4})-N_{\alpha}(nr_{3}) \bigr) \bigr) \vert A \bigr]\bigr\vert \\ & \quad{}+ \bigl\vert \mathbb{E} \bigl[\sin \bigl(\theta \bigl(N_{\alpha}(nr_{4})-N_{\alpha}(nr_{3}) \bigr) \bigr) \vert A \bigr] \bigr\vert \bigr] \bigl[ \bigl\vert \mathbb{E} \bigl[ \cos \bigl(\theta \bigl(N_{\alpha}(nr_{2})-N_{\alpha}(nr_{1}) \bigr) \bigr) \vert A \bigr] \bigr\vert \\ & \quad{}+ \bigl\vert \mathbb{E} \bigl[\sin \bigl(\theta \bigl(N_{\alpha}(nr_{1})-N_{\alpha}(nr_{1}) \bigr) \bigr) \vert A \bigr]\bigr\vert \bigr]\bigotimes _{j=1}^{4} dr_{j} \Biggr] \\ & \begin{aligned} &\leq\frac{1}{2}\mathbb{E} \Biggl[\mathbf{1}_{\{A>0\}} \frac{n^{2}}{A^{2}} \int_{[2s,2t]^{4}} \bigl\Vert \mathbb{E}e^{i\theta(N_{\alpha}(nr_{4})-N_{\alpha}(nr_{3}))}\vert A \bigr\Vert \bigl\Vert Ee^{i\theta(N_{\alpha}(nr_{2})-N_{\alpha}(nr_{1}))}\vert A \bigr\Vert \bigotimes _{j=1}^{4} dr_{j} \Biggr] \\ &\leq\frac{48}{(1-\cos\theta)^{2}}(t-s)^{2}. \end{aligned} \end{aligned}$$

Then we obtain that

$$ \mathbb{E} \bigl[I_{1,n}^{\theta}(t)-I_{1,n}^{\theta}(s) \bigr]^{4}+\mathbb {E} \bigl[I_{2,n}^{\theta}(t)-I_{2,n}^{\theta}(s) \bigr]^{4}\leq\frac{96}{(1-\cos\theta )^{2}}(t-s)^{2}=K (t-s)^{2}. $$

This completes the proof. □

### Lemma 2.2

*The set of laws of* $\{X_{n}^{\theta}\}_{n\geq1} $ *in* $\mathcal{C}([0,1], \mathbb{C})$ *is tight*.

### Proof

To prove the tightness, we have to prove that the law corresponding to the real part and the imaginary part of the process $X_{n}^{\theta}$ is tight.

In fact, it is almost sure that $Y_{n}^{\theta}(0)=0$ for all $n\geq1$. Lemma 2.1 and Theorem 12.3 of [9] show that the set of the law of the real part and the imaginary part of the process $\{Y_{n}^{\theta}\}_{n\geq1}$ is tight in $\mathcal{C}([0,1], \mathbb{R})$ for a fixed constant A. Hence, for any $\varepsilon>0$, there exists a compact set $S_{\epsilon}\subset\mathcal{C}([0,1], \mathbb{R})$ such that

4 $$ \mathbb{P} \bigl(I_{1,n}^{\theta}\in S_{\varepsilon}, n\geq1 \bigr)>1-\varepsilon/2. $$

Because *A* is a finite positive random variable, then there exists a bounded set $N_{\varepsilon}$ such that

5 $$ \mathbb{P}(A\in N_{\varepsilon})>1-\varepsilon/2. $$

Observe that the set $\Xi_{\varepsilon}:=\{af; a\in N_{\varepsilon}, f\in S_{\varepsilon}\}$ is compact in $C([0,1], \mathbb{R})$. Then, combining (4) and (5), for any $n\geq1$, we have

6 $$ \mathbb{P} \bigl(AI_{1,n}^{\theta}\in \Xi_{\varepsilon}\bigr)\geq \mathbb{P} \bigl(A\in N_{\varepsilon}, I_{1,n}^{\theta}\in S_{\varepsilon}\bigr)\geq1-\varepsilon. $$

Then, combining (3) and (6), we obtain that, for any $\varepsilon>0$, the real part of the process $X_{n}^{\theta}$ has

$$ \mathbb{P} \bigl(\operatorname{Re} \bigl(X_{n}^{\theta}\bigr) \in \Xi_{\varepsilon}\bigr)\geq 1-\varepsilon. $$

Using a similar idea, we obtain that, for any $\varepsilon>0$, the imaginary part of the process $X_{n}^{\theta}$ has

$$ \mathbb{P} \bigl(\operatorname{Im} \bigl(X_{n}^{\theta}\bigr) \in \Xi_{\varepsilon}' \bigr)\geq 1-\varepsilon. $$

This completes the proof. □

### Identification of the limit law

Let $\{P_{n_{k}}^{\theta}\}$ be a subsequence of $\{P_{n}^{\theta}\}$ weakly convergent to some probability $P^{\theta}$. If $\theta\in(0,\pi)\cup(\pi,2\pi)$, when $A>0$ is given, then the canonical process $Z=\{Z_{t}(X_{n})=:X_{n}(t)\}$ is a complex Brownian motion under the probability $P^{\theta}$, that is, the real part and the imaginary part of the process are two independent Brownian motions with respect to the probability space $(\Omega_{1}, \mathcal{F}_{1}, P_{1})$.

Using Paul Lévy’s theorem, it suffices to prove that under $P^{\theta}$, when $A>0$ is given, the real part and the imaginary part of the canonical process are both martingales with respect to the natural filtration $\{\mathcal{F}_{t}\}$, with quadratic variations $\langle\operatorname{Re}[Z],\operatorname{Re}[Z]\rangle_{t}=At$, $\langle\operatorname{Im}[Z],\operatorname{Im}[Z]\rangle_{t}=At$, and covariation $\langle\operatorname{Re}[Z],\operatorname {Im}[Z]\rangle_{t}=0$ with respect to the probability space $(\Omega_{1}, \mathcal{F}_{1}, P_{1})$.

Next, we first prove the martingale property and then prove the quadratic variations and covariation; these proofs are similar to the proof in [8]. Here, we give a sketch of the proof with some lemmas.

Let $\theta\in(0,\pi)\cup(\pi,2\pi)$, in order to prove that under $P^{\theta}$ the real and imaginary parts of the canonical process *Z* are martingales with respect to its natural filtration $\{\mathcal{F}_{t}\}$, we have to prove that for any $s_{1}\leq s_{2}\leq\cdots \leq s_{k}\leq s$, $k\geq1$ and for any bounded continuous function $\varphi:\mathbb{C}^{k}\rightarrow \mathbb{R}$ such that

7 $$ \mathbb{E}_{P^{\theta}} \bigl[\varphi(Z_{s_{1}}, \ldots,Z_{s_{k}}) \bigl(\operatorname {Re}[Z_{t}]- \operatorname{Re}[Z_{s}] \bigr) \bigr]=0, $$

and

8 $$ \mathbb{E}_{P^{\theta}} \bigl[\varphi(Z_{s_{1}}, \ldots,Z_{s_{k}}) \bigl(\operatorname {Im}[Z_{t}]- \operatorname{Im}[Z_{s}] \bigr) \bigr]=0. $$

We first consider (7).

Since $\{P_{n}^{\theta}\}$ weakly converges to $P^{\theta}$, combining Lemma 2.2, we have

$$\begin{aligned} &\lim_{n\rightarrow\infty}\mathbb{E}_{P_{n}^{\theta}} \bigl[\varphi \bigl(X_{n}^{\theta}(s_{1}),\ldots,X_{n}^{\theta}(s_{k}) \bigr) \bigl(\operatorname{Re} \bigl[X_{n}^{\theta}(t) \bigr]- \operatorname{Re} \bigl[X_{n}^{\theta}(s) \bigr] \bigr) \bigr] \\ &\quad =\mathbb{E}_{P^{\theta}} \bigl[\varphi \bigl(X_{n}^{\theta}(s_{1}), \ldots,X_{n}^{\theta}(s_{k}) \bigr) \bigl( \operatorname{Re} \bigl[X_{n}^{\theta}(t) \bigr]- \operatorname{Re} \bigl[X_{n}^{\theta}(s) \bigr] \bigr) \bigr]. \end{aligned}$$

So it suffices to prove that

$$ \mathbb{E} \bigl[\varphi \bigl(X_{n}^{\theta}(s_{1}), \ldots,X_{n}^{\theta}(s_{k}) \bigr) \bigl(I_{1,n}^{\theta}(t)-I_{1,n}^{\theta}(s) \bigr) \bigr] $$

converges to zero when $n \rightarrow\infty$. Now, we just need to prove that

$$ \lim_{n\rightarrow\infty}\bigl\Vert \mathbb{E} \bigl[\varphi \bigl(X_{n}^{\theta}(s_{1}),\ldots,X_{n}^{\theta}(s_{k}) \bigr) \bigl(I_{1,n}^{\theta}(t)-I_{1,n}^{\theta}(s) \bigr) \bigr]\bigr\Vert =0. $$

In fact

$$\begin{aligned} & \bigl\Vert \mathbb{E} \bigl[\varphi \bigl(X_{n}^{\theta}(s_{1}), \ldots,X_{n}^{\theta}(s_{k}) \bigr) \bigl(X_{n}^{\theta}(t)-X_{n}^{\theta}(s) \bigr) \bigr]\bigr\Vert \\ &\quad = \biggl\Vert \mathbb{E} \bigl[\varphi \bigl(X_{n}^{\theta}(s_{1}), \ldots ,X_{n}^{\theta}(s_{k}) \bigr)e^{i\theta N_{\alpha}(2ns)} \bigr] \mathbb{E} \biggl[ \sqrt{n} \int_{2s}^{2t} e^{i\theta(N_{\alpha}(nr)-N_{\alpha}(2ns))}\,dr \biggr] \biggr\Vert \\ &\quad \leq K \biggl\Vert \mathbb{E} \biggl[ \sqrt{n} \int_{2s}^{2t} \mathbb{E} \bigl[e^{i\theta(N_{\alpha}(nr)-N_{\alpha}(2ns))} \bigr]\,dr \biggr] \biggr\Vert \\ &\quad \leq K \sqrt{n} \int_{2s}^{2t} \bigl\Vert \mathbb{ \mathbb{E}} \bigl[e^{i\theta(N_{\alpha}(nr)-N_{\alpha}(2ns))} \bigr] \bigr\Vert \,dr \\ &\quad =K \sqrt{n} \int_{2s}^{2t} e^{-\frac{n}{A}(r-2s)(1-\cos\theta)}\,dr \\ &\quad =K \sqrt{\frac{A}{ {n}}}\frac{ 1-e^{-\frac{n}{A}(2t-2s)(1-\cos\theta )} }{ 1-\cos\theta} \\ &\quad \rightarrow0,\quad n\rightarrow\infty. \end{aligned}$$

Using the same idea of proof (7), we can obtain the proof of (8).

We give the following auxiliary lemma.

### Lemma 2.3

*Let* $\theta\in(0,\pi)\cup(\pi,2\pi)$. *Consider the natural filtration* $\{\mathcal{F}_{t}^{n,\theta}\}$ *of the process* $Y_{n}^{\theta}$. *Then*, *for any* $s< t$ *and for any real* $\mathcal{F}_{s}^{n,\theta}$-*measurable and bounded random variable* *H*, *if* $A>0$ *is given*, *we have*

- $\lim_{n\rightarrow\infty} \mathbb{E} [ {n}\int_{2s}^{2t} \int_{2s}^{r_{2}} e^{i\theta(N_{\alpha}(nr_{2})-N_{\alpha}(nr_{1}))}\,dr_{1}\,dr_{2} ] =A(t-s) (1+i\frac{\sin\theta}{1-\cos\theta} )$;
- $\lim_{n\rightarrow\infty} \Vert \mathbb{E} [ {n}\int_{2s}^{2t} \int_{2s}^{r_{2}} [e^{i\theta(N_{\alpha}(nr_{2})+N_{\alpha}(nr_{1}))}H ]\,dr_{1}\,dr_{2} ] \Vert =0$.

### Proof

We first prove (a). In fact

$$\begin{aligned} &\mathbb{E} \biggl[ {n} \int_{2s}^{2t} \int_{2s}^{r_{2}} e^{i\theta(N_{\alpha}(nr_{2})-N_{\alpha}(nr_{1}))}\,dr_{1} \,dr_{2} \biggr] \\ &\quad = {n} \int_{2s}^{2t} \int_{2s}^{r_{2}} e^{-\frac{A}{n}(r_{2}-r_{1}) (1-e^{i\theta} )}\,dr_{1} \,dr_{2} \\ &\quad = A(t-s) \biggl(1+i\frac{\sin\theta}{1-\cos\theta} \biggr) +o \biggl(\frac{A}{n} \biggr) , \end{aligned}$$

then

$$ \lim_{n\rightarrow\infty} \mathbb{E} \biggl[ {n} \int_{2s}^{2t} \int_{2s}^{r_{2}} e^{i\theta(N_{\alpha}(nr_{2})-N_{\alpha}(nr_{1}))}\,dr_{1} \,dr_{2} \biggr] =A(t-s) \biggl(1+i\frac{\sin\theta}{1-\cos\theta} \biggr). $$

Now we prove (b). Using the fact that the Poisson process has independent increments, we can obtain

$$\begin{aligned} & \biggl\Vert \mathbb{E} \biggl[ {n} \int_{2s}^{2t} \int_{2s}^{r_{2}} \bigl[e^{i\theta(N_{\alpha}(nr_{2})+N_{\alpha}(nr_{1}))}H \bigr] \,dr_{1}\,dr_{2} \biggr] \biggr\Vert \\ &\quad = \biggl\Vert {n} \int_{2s}^{2t} \int_{2s}^{r_{2}} E \bigl[e^{i\theta(N_{\alpha}(nr_{2})-N_{\alpha}(nr_{1}))+ i2\theta \bigl(N_{\alpha}(nr_{1})-N_{\alpha}(2ns) \bigr) +i2\theta N_{\alpha}(2ns) }H \bigr] \,dr_{1} \,dr_{2} \biggr\Vert \\ &\quad = \biggl\Vert {n} \int_{2s}^{2t} \int_{2s}^{r_{2}} \bigl[Ee^{i\theta(N_{\alpha}(nr_{2})-N_{\alpha}(nr_{1}))} \bigr] \bigl[Ee^{i2\theta(N_{\alpha}(nr_{1})-N_{\alpha}(2ns))} \bigr] \bigl[Ee^{i2\theta N_{\alpha}(2ns) }H \bigr] \,dr_{1}\,dr_{2} \biggr\Vert \\ &\quad \leq K {n} \int_{2s}^{2t} \int_{2s}^{r_{2}} \bigl\Vert e^{-\frac{n}{A}(r_{2}-r_{1})(1-e^{i\theta })} \bigr\Vert \bigl\Vert e^{-\frac{n}{A}(r_{1}-2s)(1-e^{i2\theta})} \bigr\Vert \,dr_{1} \,dr_{2} \\ &\quad =K {n} \int_{2s}^{2t} \int_{2s}^{r_{2}} e^{-\frac{n}{A}(r_{2}-r_{1})(1-\cos\theta)} e^{-\frac {n}{A}(r_{1}-2s)(1-\cos(2\theta))} \,dr_{1}\,dr_{2} \\ &\quad = {\frac{K Ae^{\frac{2ns}{A}(1-\cos\theta)}}{\cos\theta-\cos(2\theta )}} \int_{2s}^{2t} e^{-\frac{n}{A}(1-\cos\theta)r_{2}}\,dr_{2} \\ &\qquad{}- {\frac{K Ae^{\frac{2ns}{A}(1-\cos(2\theta))}}{\cos\theta-\cos (2\theta)}} \int_{2s}^{2t}e^{-\frac{n}{A}(1-\cos(2\theta))r_{2}}\,dr_{2} \\ &\begin{aligned} &\quad = {\frac{K A^{2}(1-\cos\theta)}{n(\cos\theta-\cos(2\theta))}} - {\frac{K A^{2} (1-e^{-\frac{2n(t-s)}{A}(1-\cos(2\theta))} )}{n(\cos\theta-\cos(2\theta))(1-\cos(2\theta))}} \\ &\quad \rightarrow 0,\quad n \rightarrow\infty. \end{aligned} \end{aligned}$$

This completes the proof. □

### Lemma 2.4

*Consider* $\{P_{n}^{\theta}\}$ *the laws on* $C([0,T],\mathbb{C})$ *of the processes* $X_{n}^{\theta}$, *and assume that* $\{P_{n_{k}}^{\theta}\}$ *is a subsequence weakly convergent to* $P^{\theta}$. *Let* *Z* *be the canonical process*, *and let* $\{F_{t}\}$ *be its natural filtration*. *Then*, *if* *A* *is given*, *under* $P^{\theta}$, *when* $\theta\in (0,\pi)\cup(\pi,2\pi)$, *we have the quadratic variations* $\langle\operatorname{Re}[Z],\operatorname{Re}[Z]\rangle_{t}=At$, $\langle\operatorname{Im}[Z],\operatorname{Im}[Z]\rangle_{t}=At$, *and covariation* $\langle\operatorname{Re}[Z],\operatorname {Im}[Z]\rangle_{t}=0$ *with respect to the probability space* $(\Omega_{1}, \mathcal{F}_{1}, P_{1})$.

### Proof

Let $\theta\in(0,\pi)\cup(\pi,2\pi)$, *A* is given. We will prove that $\langle\operatorname{Re}[Z],\operatorname{Re}[Z]\rangle_{t}=At$, $\langle\operatorname{Im}[Z],\operatorname{Im}[Z]\rangle_{t}=At$, $\langle\operatorname{Re}[Z],\operatorname{Im}[Z]\rangle_{t}=0$. It is enough to prove that for any $s_{1}\leq s_{2}\leq\cdots \leq s_{k}\leq s$, $k\geq1$ and for any bounded continuous function $\varphi:\mathbb{C}^{k}\rightarrow \mathbb{R}$, such that

9 $$\begin{aligned}& \lim_{n\rightarrow\infty}\mathbb{E} \bigl[\varphi \bigl(X_{n}^{\theta}(s_{1}),\ldots ,X_{n}^{\theta}(s_{k}) \bigr) \bigl( \bigl(I_{1,n}^{\theta}(t)-I_{1,n}^{\theta}(s) \bigr)^{2}-A(t-s) \bigr) \bigr]=0, \end{aligned}$$

10 $$\begin{aligned}& \lim_{n\rightarrow\infty}\mathbb{E} \bigl[\varphi \bigl(X_{n}^{\theta}(s_{1}),\ldots ,X_{n}^{\theta}(s_{k}) \bigr) \bigl( \bigl(I_{2,n}^{\theta}(t)-I_{2,n}^{\theta}(s) \bigr)^{2}-A(t-s) \bigr) \bigr]=0, \end{aligned}$$

11 $$\begin{aligned}& \lim_{n\rightarrow\infty}\mathbb{E} \bigl[\varphi \bigl(X_{n}^{\theta}(s_{1}),\ldots ,X_{n}^{\theta}(s_{k}) \bigr) \bigl( \bigl(I_{1,n}^{\theta}(t)-I_{1,n}^{\theta}(s) \bigr) \bigl(I_{2,n}^{\theta}(t)-I_{2,n}^{\theta}(s) \bigr) \bigr) \bigr]=0. \end{aligned}$$

In fact

$$\begin{aligned} &\mathbb{E} \biggl[\varphi \bigl(X_{n}^{\theta}(s_{1}), \ldots,X_{n}^{\theta}(s_{k}) \bigr) \biggl( \sqrt{n} \int_{2s}^{2t}\cos \bigl(\theta N_{\alpha}(nr) \bigr)\,dr \biggr)^{2} \biggr] \\ &\quad =2\mathbb{E} \biggl[\varphi \bigl(X_{n}^{\theta}(s_{1}), \ldots,X_{n}^{\theta}(s_{k}) \bigr) \biggl( n \int_{2s}^{2t} \int_{2s}^{r_{2}} \cos \bigl(\theta N_{\alpha}(nr_{1}) \bigr)\cos \bigl(\theta N_{\alpha}(nr_{2}) \bigr) \,dr_{1}\,dr_{2} \biggr) \biggr] \\ &\quad =\mathbb{E} \biggl[\varphi \bigl(X_{n}^{\theta}(s_{1}), \ldots,X_{n}^{\theta}(s_{k}) \bigr) \biggl( n \int_{2s}^{2t} \int_{2s}^{r_{2}} \cos \bigl(\theta \bigl(N_{\alpha}(nr_{2})-N_{\alpha}(nr_{1}) \bigr) \bigr)\\ &\qquad {}+ \cos \bigl(\theta \bigl(N_{\alpha}(nr_{2})+N_{\alpha}(nr_{1}) \bigr) \bigr)\,dr_{1}\,dr_{2} \biggr) \biggr] \\ &\quad = n \int_{2s}^{2t} \int_{2s}^{r_{2}} \cos \bigl(\theta \bigl(N_{\alpha}(nr_{2})-N_{\alpha}(nr_{1}) \bigr) \bigr)\,dr_{1}\,dr_{2} E \bigl[\varphi \bigl(X_{n}^{\theta}(s_{1}), \ldots,X_{n}^{\theta}(s_{k}) \bigr) \bigr] \\ &\qquad{} + \mathbb{E} \biggl[ \int_{2s}^{2t} \int_{2s}^{r_{2}} \varphi \bigl(X_{n}^{\theta}(s_{1}), \ldots,X_{n}^{\theta}(s_{k}) \bigr) \cos \bigl(\theta \bigl(N_{\alpha}(nr_{2})+N_{\alpha}(nr_{1}) \bigr) \bigr)\,dr_{1}\,dr_{2} \biggr] \\ &\quad =\operatorname{Re} \biggl\{ n \int_{2s}^{2t} \int_{2s}^{r_{2}} e^{\theta(N_{\alpha}(nr_{2})-N_{\alpha}(nr_{1}))}\,dr_{1} \,dr_{2} \biggr\} E \bigl[\varphi \bigl(X_{n}^{\theta}(s_{1}), \ldots,X_{n}^{\theta}(s_{k}) \bigr) \bigr] \\ &\qquad{} + \operatorname{Re} \biggl\{ \mathbb{E} \biggl[ n \int_{2s}^{2t} \int_{2s}^{r_{2}} \varphi \bigl(X_{n}^{\theta}(s_{1}), \ldots,X_{n}^{\theta}(s_{k}) \bigr) e^{\theta(N_{\alpha}(nr_{2})+N_{\alpha}(nr_{1}))} \,dr_{1}\,dr_{2} \biggr] \biggr\} . \end{aligned}$$

Combining Lemma 2.3 and that $P_{n}^{\theta}$ converges weakly to $P^{\theta}$, we can obtain that when $n\rightarrow\infty$, above two integrals converge to $A(t-s)E[\varphi(Z_{s_{1}},\ldots,Z_{s_{k}})]$. This completes the proof of (9).

Similarly, when *n* tends to infinity, we obtain the expression

$$ \mathbb{E} \bigl[\varphi \bigl(X_{n}^{\theta}(s_{1}), \ldots,X_{n}^{\theta}(s_{k}) \bigr) \bigl(I_{2,n}^{\theta}(t)-I_{2,n}^{\theta}(s) \bigr)^{2} \bigr] $$

converges to $A(t-s)E[\varphi(Z_{s_{1}},\ldots,Z_{s_{k}})]$. This completes the proof of (10).

Now, we prove (11).

$$\begin{aligned} &\mathbb{E} \bigl[\varphi \bigl(X_{n}^{\theta}(s_{1}), \ldots,X_{n}^{\theta}(s_{k}) \bigr) \bigl( \bigl(I_{1,n}^{\theta}(t)-I_{1,n}^{\theta}(s) \bigr) \bigl(I_{2,n}^{\theta}(t)-I_{2,n}^{\theta}(s) \bigr) \bigr) \bigr] \\ &\quad =\mathbb{E} \biggl[\varphi \bigl(X_{n}^{\theta}(s_{1}), \ldots,X_{n}^{\theta}(s_{k}) \bigr) \biggl( n \int_{2s}^{2t} \cos \bigl(\theta \bigl(N_{\alpha}(nr) \bigr) \bigr)\,dr \int_{2s}^{2t} \sin \bigl(\theta \bigl(N_{\alpha}(nr) \bigr) \bigr) \,dr \biggr) \biggr] \\ &\quad =\mathbb{E} \biggl[ {n} \int_{2s}^{2t} \int_{2s}^{r_{2}} \bigl(\varphi \bigl(X_{n}^{\theta}(s_{1}), \ldots,X_{n}^{\theta}(s_{k}) \bigr) \bigr) \cos \bigl( \theta \bigl(N_{\alpha}(nr_{1}) \bigr) \bigr)\sin \bigl(\theta \bigl(N_{\alpha}(nr_{2}) \bigr) \bigr)\,dr_{1} \,dr_{2} \biggr] \\ &\qquad{}+\mathbb{E} \biggl[n \int_{2s}^{2t} \int_{2s}^{r_{2}} \bigl(\varphi \bigl(X_{n}^{\theta}(s_{1}), \ldots,X_{n}^{\theta}(s_{k}) \bigr) \bigr) \sin \bigl( \theta \bigl(N_{\alpha}(nr_{1}) \bigr) \bigr)\cos \bigl(\theta \bigl(N_{\alpha}(nr_{2}) \bigr) \bigr)\,dr_{1} \,dr_{2} \biggr] \\ &\quad =\mathbb{E} \biggl[ {n} \int_{2s}^{2t} \int_{2s}^{r_{2}} \bigl(\varphi \bigl(X_{n}^{\theta}(s_{1}), \ldots,X_{n}^{\theta}(s_{k}) \bigr) \bigr)\sin \bigl( \theta \bigl(N_{\alpha}(nr_{1})+N_{\alpha}(nr_{2}) \bigr) \bigr)\,dr_{1}\,dr_{2} \biggr] \\ &\quad =\operatorname{Im} \biggl[\mathbb{E} \biggl[ {n} \int_{2s}^{2t} \int_{2s}^{r_{2}} \bigl(\varphi \bigl(X_{n}^{\theta}(s_{1}), \ldots,X_{n}^{\theta}(s_{k}) \bigr) \bigr)e^{\theta(N_{\alpha}(nr_{1})+N_{\alpha}(nr_{2}))}\,dr_{1}\,dr_{2} \biggr] \biggr]. \end{aligned}$$

From (2) of Lemma 2.3, when *n* tends to infinity, we have

$$\mathbb{E} \bigl[\varphi \bigl(X_{n}^{\theta}(s_{1}), \ldots,X_{n}^{\theta}(s_{k}) \bigr) \bigl( \bigl(I_{1,n}^{\theta}(t)-I_{1,n}^{\theta}(s) \bigr) \bigl(I_{2,n}^{\theta}(t)-I_{2,n}^{\theta}(s) \bigr) \bigr) \bigr] \rightarrow0. $$

This completes the lemma. □

### Lemma 2.5

*If* $\theta \in(0,\pi)\cup(\pi,2\pi)$, $A>0$ *is given*. *For any* $T>0$, *when* $n\rightarrow\infty$, *the real part and the imaginary part of* $X_{n}^{\theta}$ *converge weakly in* $\mathcal{C}([0,T], \mathbb{R})$, *with respect to* $(\Omega_{1}, \mathcal{F}_{1}, P_{1})$, *to independent zero mean and variance* *At* *Brownian motions* $B_{1}(t)$ *and* $B_{2}(t)$, *respectively*.

### Proof

When *A* is given, $N_{\alpha}(t)$ is the Poisson process with non-random intensity $\frac{1}{A}$ with respect to $(\Omega_{1}, \mathcal{F}_{1}, P_{1})$. Thus, for any $T>0$, by Lemma 2.4, the real part and the imaginary part of $X_{n}^{\theta}$ converge weakly in $\mathcal{C}([0,T], \mathbb{R})$, with respect to $(\Omega_{1}, \mathcal{F}_{1}, P_{1})$, to two independent zero mean and variance *At* Brownian motions $B_{1}(t)$ and $B_{2}(t)$, respectively. □

### Lemma 2.6

*If* $\theta \in(0,\pi)\cup(\pi,2\pi)$. *When* $n\rightarrow\infty$, *the real part and the imaginary part of* $X_{n}^{\theta}$ *converge in law to sub*-*Gaussian processes* $\{\sqrt{A_{1}}G_{1}(t)\}$, $\{\sqrt{A_{1}}G_{2}(t)\}$, $t\in[0,1]$, *respectively*, *where* $\{G_{1}(t)\}$, $\{G_{2}(t)\}$, $t\in[0,1]$ *are independent copies of a standard Brownian motion* $G(t)$, $A_{1}$ *is a random variable with the same law as* *A*.

### Proof

Combining Lemma 2.5, the proof is similar to the proof of Theorem 1 of [7]. □

### Proof of Theorem 2.1

Combining Lemma 2.2, Lemma 2.6 and Corollary 2.6.4 of [1], we can obtain the conclusion. □

## Conclusions

We prove that an isotropic complex symmetric *α*-stable random measure ($0 < \alpha< 2$) can be approximated by a complex process constructed by integrals based on the Poisson process with random intensity, which gives a new method to construct complex-valued random measure.
